# Correlation Between Coronavirus Disease 2019 Severity and Noninvasive Assessment of Liver Fibrosis in Patients with Metabolic Dysfunction-Associated Fatty Liver Disease

**DOI:** 10.5152/tjg.2023.23004

**Published:** 2023-12-01

**Authors:** Nuttapat Tungtrongchitr, Nantaporn Srivanitchapoom, Pornrujee Hirunpat, Somnuek Sungkanuparph

**Affiliations:** Chakri Naruebodindra Medical Institute, Ramathibodi Hospital, Mahidol University Faculty of Medicine, Bangkok, Thailand

**Keywords:** MAFLD, COVID-19, fibrosis 8 score, fibrosis 4 score, nonalcoholic fatty liver disease fibrosis score

## Abstract

**Background/Aims:**

Metabolic dysfunction-associated fatty liver disease is a crucial global health concern. Studies have shown that metabolic dysfunction-associated fatty liver disease patients are at higher risk of severe coronavirus disease 2019. However, there are no precise measures of the correlation between the degree of metabolic dysfunction-associated fatty liver disease fibrosis and coronavirus disease 2019 severity. This study evaluated the association between metabolic dysfunction-associated fatty liver disease with varying degrees of fibrosis and coronavirus disease 2019 prognosis.

**Materials and Methods:**

All hospitalized coronavirus disease 2019 patients who had liver steatosis as determined by computed tomography scan were included. Metabolic dysfunction-associated fatty liver disease was diagnosed in accordance with international consensus criteria. Liver fibrosis was assessed using the nonalcoholic fatty liver disease fibrosis score, FIB-4 and FIB-8 indexes. Coronavirus disease 2019 severity was defined using World Health Organization criteria. Logistic regression was used to determine the associations between varying degrees of fibrosis and the severity of coronavirus disease 2019.

**Results:**

A total of 996 confirmed hospitalized coronavirus disease 2019 cases with complete data were reviewed; of these, 296 (29.7%) cases of metabolic dysfunction-associated fatty liver disease were diagnosed. Metabolic dysfunction-associated fatty liver disease patients with any fibrotic state had more severe coronavirus disease 2019 than nonmetabolic dysfunction-associated fatty liver disease patients (adjusted odds ratio 1.912, 95% CI 1.363-2.684; *P *< .05). Multiple logistic regression analysis showed that metabolic dysfunction-associated fatty liver disease patients with significant fibrosis according to the FIB-8 score were more likely to have severe coronavirus disease 2019 (adjusted odds ratio 5.458, 95% CI 1.481-20.110; *P* < .05).

**Conclusion:**

The presence of metabolic dysfunction-associated fatty liver disease in hospitalized coronavirus disease 2019 patients strongly correlated with the severity of coronavirus disease 2019. The hepatic FIB-8 index appears to provide the best prognostic value among the fibrosis scores in metabolic dysfunction-associated fatty liver disease patients with coronavirus disease 2019.

**Main Points**

- The presence of metabolic dysfunction-associated fatty liver disease (MAFLD) in hospitalized coronavirus disease 2019 (COVID-19) patients strongly correlated with the severity of COVID-19.
- In the MAFLD group, we encourage to evaluate fibrotic status using various method to determine the prognosis.
- The hepatic FIB-8 index trends to be more accurate than the other scores in predicting the COVID-19 prognosis in MAFLD patients who have significant fibrosis.

## Introduction

Metabolic dysfunction-associated fatty liver disease (MAFLD), formerly known as nonalcoholic fatty liver disease (NAFLD), is a major health concern and is associated with socioeconomic problems worldwide.^1, 2^ The global prevalence of MAFLD comprises greater than one-fourth people worldwide but depends on age, sex, ethnicity, and diet, which varies among regions.^2, 3^ Metabolic dysfunction-associated fatty liver disease is an important risk factor for hepatocellular carcinoma and the associated morbidity and mortality.

The new MAFLD criteria proposed by an international consensus in 2020 are the appearance of hepatic steatosis in the absence of significant ongoing alcohol drinking or other causes of chronic liver disease concomitant with one of the following: type 2 diabetes mellitus (T2DM), overweight/obesity or at least 2 metabolic risk abnormalities.^4^ Hepatic steatosis is usually found incidentally in patients who undergo chest or abdominal computed tomography (CT).^5, 6^

Severe acute respiratory syndrome coronavirus 2 (SARS-CoV-2) caused coronavirus disease resulting in pandemic in 2020.^7^ Coronavirus disease 2019 (COVID-19) patients who died were often obese and had additional metabolic risk factors such as hypertension, diabetes, dyslipidemia, and MAFLD, which may have contributed to their greater risk of COVID-19.^8-10^ These patients often underwent a CT chest scan that included the upper abdomen to evaluate pulmonary involvement during admission. At this time, hepatic steatosis may have been incidentally detected. Metabolic dysfunction-associated fatty liver disease was diagnosed in these patients during admission. To evaluate the severity of liver fibrosis, which can help to determine prognosis, scoring systems (e.g., NAFLD fibrosis score (NFS), fibrosis-4 score (FIB-4), and the newly proposed fibrosis-8 score (FIB-8)) can be used as noninvasive methods instead of liver biopsy.^11, 12^

Recent studies have shown that approximately 30% of COVID-19 patients have MAFLD.^13^ The MAFLD patients have a greater risk of COVID-19 progression, a greater likelihood of transaminitis upon admission.^14^ However, the prognosis of MAFLD is altered by the degree of liver fibrosis, which has not been investigated in association with COVID-19 severity. Therefore, we studied the correlation between noninvasive fibrosis scores and outcomes in hospitalized COVID-19 patients in our center.

## Materials and Methods

This retrospective study was conducted at the referral tertiary care hospital. The electronic medical records from April 2021 to December 2021 were reviewed. Since this report is a retrospective study, informed consent was not acquired from the patients. The study was approved by the Human Research Ethics Committee of Mahidol University Faculty of Medicine, Ramathibodi Hospital (MURA2022/15). A total of 1014 hospitalized patients older than 18 years with a COVID-19 infection confirmed by real-time polymerase chain reaction were included in this study. The patients with previously known chronic liver disease and those who were positive for viral hepatitis had a history of hepatobiliary cancers (e.g., hepatocellular carcinoma, cholangiocarcinoma, or liver metastasis), or who had excessive alcohol intake were excluded from this report.

All admitted patients underwent the following blood tests: complete blood count, glucose, creatinine, serum electrolytes, liver function, and lipid profile. Human immunodeficiency virus and hepatitis B and C tests were also performed and collected at the admission time. During admission, all of the patients were scanned with the same CT scanner (uCT 530, United Imaging). Computed tomography was conducted with the following criterions: 120 kVp, automatic mAs, 1.1250 pitch, 0.5 s rotation time, slice collimation 0.55 mm × 40. An inclusion criterion was that the noncontrast chest CT scans had to include the upper abdomen, which extends caudally, so at least the right portal vein branch and the splenic hilum could be visualized. Hepatic steatosis was diagnosed using the following criteria: relative hypoattenuation: liver attenuation more than 10 HU less than that of the spleen, absolute low attenuation: liver attenuation less than 40 HU.^15^ By using this criteria, previous reports showed sensitivity and specificity ranging between 46%-72% and 88%-95%, respectively.^6^

COVID-19 severity was classified in accordance with the WHO criteria as severe and nonsevere illness.^16^ Hospitalized COVID-19 patients who were diagnosed with MAFLD were categorized as having low, intermediate, or high probability of significant fibrosis using the NFS, the fibrosis-4 index (FIB-4), and the fibrosis-8 index (FIB-8). The noninvasive liver fibrosis tests used the same laboratory results as above. Low probability of significant fibrosis was classified as NFS <−1.455, FIB-4 <1.45, and FIB-8 <0.88. High probability of significant fibrosis was classified as NFS >0.675, FIB-4 >3.25, and FIB-8 >1.77. Intermediate probability was defined as the values between the low and high probability ranges.

### Statistical Analysis

The baseline characteristics of the patients including demographic data, underlying diseases, and lab tests are presented as the median ± interquartile range (IQR) for continuous variables and as the frequency (%) for categorical variables. The chi-square test was used to analyze categorical variables. The Mann–Whitney *U*-test was used to compare medians between 2 groups. Risk factors for severe COVID-19 illness among patients were analyzed using univariate logistic regression and presented as the odds ratio (OR) and 95% confidence interval (CI). Risk factors identified by univariate analysis (*P* < .2) were put into the multivariate analysis. The correlation between risk factors were determined using Spearman correlation (rho). Statistical significance was considered as *P* < .05, and all reported probability tests were 2 sided. The statistical analyses were conducted using Statistical Package for the Social Sciences version 18.0 (SPSS Inc.; Chicago, IL, USA).

## Results

The electronic medical records from April 2021 to December 2021 were reviewed. Over the study period, 1014 patients were diagnosed with COVID-19 infection in our center, from which 53 were excluded for missing data or no CT scan. Thus, we included and collected data from 996 patients. Overall, 296 patients (29.7%) had fatty liver by CT scan assessment and were diagnosed with MAFLD in accordance with the recent international consensus.

The baseline demographic data of the MAFLD and non-MAFLD patients are shown in Table 1. A majority of the MAFLD patients were women (54.1%), with a median age of 47 years. The MAFLD patients tended to be more obese and higher BMI than the non-MAFLD patients. The prevalence of diabetes and hypertension in MAFLD patients was 24.3% and 27.8%, respectively. The lipid parameters (cholesterol, triglycerides, and low-density lipoprotein) in the MAFLD patients were significantly higher than in the patients without MAFLD (all *P* < .05).

Next, we determined the associations between risk factors and disease severity. In the univariate analysis, older age, male sex, high BMI, alcohol consumption, smoking, diabetes, hypertension, low platelet count, and high levels of cholesterol, triglycerides, low-density lipoprotein (LDL), and transaminase were significantly associated with a higher risk of severe COVID-19. Metabolic dysfunction-associated fatty liver disease was strongly correlated with BMI (rho = 0.402, *P *< .001), diabetes (rho = 0.115, *P *< .001), hypertension (rho = 0.146, *P* = .014), cholesterol (rho = 0.156, *P* < .001), LDL (rho = 0.230, *P < *.001), triglycerides (rho = 0.168, *P* < .001), and alanine aminotransferase (ALT) (rho = 0.238, *P* < .001). The correlations between risk factors are presented in Supplementary Table 1.

The multiple logistic regression revealed that MAFLD patients tended to have more severe COVID-19 than the non-MAFLD patients (adjusted OR 1.912, 95% CI 1.363-2.684; *P *< 0.05). Older age, low albumin levels, and high globulin levels were also significantly associated with severe COVID-19 (Table 2).

**Figure 1.**
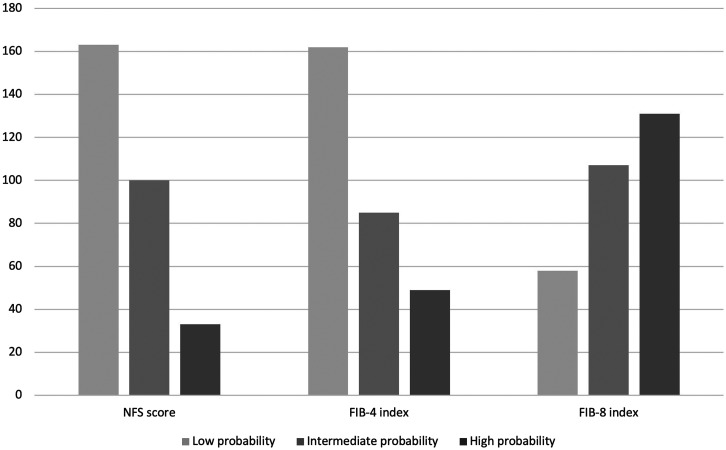
Demographic data of MAFLD patients using each fibrosis score.

After that, the MAFLD patients were classified into 3 groups depended on the probability of liver fibrosis using the NFS, FIB-4, and FIB-8. The variability in the fibrosis scores among the 3 groups is shown in Figure 1.

We determined the associations between the risk factors in MAFLD patients and the severity of COVID-19. In univariate analysis, older age, male sex, BMI, diabetes, hypertension, low albumin levels, high globulin levels, and intermediate to high probability of liver fibrosis according to the NFS, FIB-4, and FIB-8 were significantly associated with a higher risk of severe COVID-19. The multivariate analysis showed that older age, low albumin levels, and high globulin levels were significantly associated with severe COVID-19. Interestingly, MAFLD patients with an intermediate or high probability of liver fibrosis according to the FIB-8 index were significantly associated with severe disease (adjusted OR 3.553, 95% CI 1.143-11.043; *P* < .05 and 5.458, 95% CI 1.481-20.110; *P* < .05, respectively). The NFS and FIB-4 were not significantly associated with severe disease after adjusting for the other risk factors (Table 3).

## Discussion

This study was conducted at a referral tertiary care hospital that treated patients with COVID-19 infection. We evaluated the associations between CT-diagnosed MAFLD with varying degrees of fibrosis and the severity of hospitalized COVID-19 patients. Overall, 29.7% of the hospitalized COVID-19 patients were diagnosed with MAFLD, which was similar to a recent study that identified MAFLD in 28%-38.5% of hospitalized COVID-19 patients.^17^ In our study, patients with MAFLD were predominantly younger (median age of 47 years) and women. The metabolic comorbidity of T2DM was found in 24.3% of the COVID-19 patients, and hypertension was found in 27.8%, which were slightly different rates from those found in similar previous studies.^18, 19^ In the same way, MAFLD patient in our study trend to be higher BMI and obese than those without MAFLD.

Our study found that the patients with MAFLD had more severe COVID-19 illness than patients without MAFLD (adjusted OR 1.912, *P *< .05). These results are consistent with other studies that have found that compared with non-MAFLD patients, those with MAFLD have a higher risk of COVID-19 severity and transaminitis.^20, 21^

The microvascular damage and impaired tissue repair were suggested in the MAFLD and other metabolic diseases.^22^ Previous study proposed that these diseases increased the production of proinflammatory cytokines. Impaired innate immunity was demonstrated by disproportion between subtypes of macrophages.^23^ Our report supports the evidence of lack immunity resulting in progression disease severity.

The prognosis of MAFLD is altered by the severity of liver fibrosis rather than by the appearance of liver steatosis.^24^ In a previous retrospective study, the presence of fibrosis was strongly correlated with an increased risk for intubation, development of renal failure, and greater mortality in hospitalized COVID-19 patients.^25^ Therefore, we investigated the association between COVID-19 severity and MAFLD with significant fibrosis as assessed by various scoring systems.

In a previous study, MAFLD patients with NFS determined high probability of liver fibrosis were found to be at greater risk of severe COVID-19.^26^ The FIB-4 index is also strongly correlated with mortality in COVID-19 patients.^27^ The FIB-8 index is a newly proposed score that can be used to predict significant fibrosis and may be more accurate than the NFS.^12^ Our study determined the fibrosis stage using the NFS, FIB-4 index, and the newly proposed FIB-8 index.

The most interesting finding was that the probability of fibrosis as assessed by the FIB-8 was useful in determining the COVID-19 prognosis. Metabolic dysfunction-associated fatty liver disease patients with an intermediate or high probability of fibrosis according to the FIB-8 were significantly associated with severe COVID-19 (adjusted ORs 3.553 and 5.458, respectively, *P* < .05). In contrast to earlier findings, the NFS and FIB-4 index were not significantly associated with COVID-19 severity.

In our study, MAFLD patients with high levels of globulin were significantly associated with severe COVID-19 (adjusted OR 2.798, 95% CI 1.126-6.951; *P* < .05). Our results provide further support for the hypothesis that the globulin level may be an important indicator of COVID-19 prognosis in MAFLD patients who have significant fibrosis.

Low albumin levels were still significantly associated with severe COVID-19 after the multivariate analysis among hospitalized COVID-19 patients with or without MAFLD. This finding also accords with our earlier observations that low albumin was associated with COVID-19 severity.^28^ These results suggest that albumin levels are supposed to be assessed in hospitalized COVID-19 patients, especially in the MAFLD population.

The albumin and globulin level are the factors used to calculate the FIB-8 index; thus, the lower albumin and higher globulin level will result in the higher FIB-8 index. It can thus be suggested that FIB-8 is more accurate than the other scores in predicting the COVID-19 prognosis in MAFLD patients who have significant fibrosis.

This study had some limitations. First, using CT scanning and noninvasive scores to diagnose liver steatosis and the fibrotic state may not have been the best modalities. However, owing to the COVID-19 situation, to prevent patient contact, we could only use basic methods. Therefore, additional assessment methods such as transient elastography were not conducted. Second, other prognostic markers that can be used to assess the COVID-19 prognosis, such as D-dimer and lactate dehydrogenase levels, were not determined.

The main strength of our study is that it was conducted in a referral tertiary care hospital that was one of the large COVID-19 treatment centers during the coronavirus pandemic in Thailand. Additionally, we used the recently developed FIB-8 index to assess the fibrotic state to predict COVID-19 severity among MAFLD patients. The FIB-8 index was compared to the NFS and FIB-4 index, which were described to be significant risk factors of severe COVID-19 infection. Further research should be undertaken to investigate the correlations between COVID-19 prognosis and significant fibrosis among MAFLD patients using various scoring systems. However, the FIB-8 index appears to have considerable prognostic value.

## Conclusion

The presence of MAFLD in hospitalized COVID-19 patients strongly correlated with the severity of COVID-19. The hepatic FIB-8 index appears to provide the best prognostic value among fibrosis scores in MAFLD patients with COVID-19 infection.

## Figures and Tables

**Table 1.** Baseline Patient Characteristics According to the Presence or Absence of MAFLD

	All Patients (n = 996)	MAFLD (n= 296)	Non-MAFLD (n = 700)	*P*
Age (years), [median (IQR)]	49 (33-62)	47 (15-87)	50 (16-94)	.183
Male, N (%)	431 (43.3%)	136 (45.9%)	295 (42.1%)	.268
Weight (kg), [median (IQR)]	68 (58-81)	80 (38.5-148)	64.4 (36.5-120)	<.001
Height (m), [median (IQR)]	1.61 (1.55-1.7)	1.63 (1.35-1.90)	1.6 (1.40-1.89)	.006
BMI (kg/m^2^), [median (IQR)]	25.91 (22.31-30.47)	29.66 (26.17-34.14)	24.58 (21.33-28.12)	<.001
Alcohol, n (%)	230 (23.1%)	66 (22.2%)	164 (23.4%)	.699
Smoking, n (%)	128 (12.9%)	43 (14.5%)	85 (12.1%)	.295
DM, n (%)	175 (17.6%)	72 (24.3%)	103 (14.7%)	<.001
HT, n (%)	304 (30.5%)	100 (33.8%)	204 (29.1%)	.146
SBP (mmHg), [median (IQR)]	130 (118-143)	133 (121-146)	128 (118-140)	<.001
DBP (mmHg), [median (IQR)]	83 (76-91)	85 (78-94)	82 (75-90)	<.001
Initial blood tests				
Cholesterol (mg/dL), [median (IQR)]	184 (161-203)	188 (168-230)	178 (155.25-198.75)	<.001
Triglyceride (mg/dL), [median (IQR)]	113 (84-150)	124 (95-165)	103 (80-142)	<.001
LDL (mg/dL), [median (IQR)]	115 (92.8-137.95)	126.4 (106.4-159.8)	108.8 (87.2-130.6)	<.001
HDL (mg/dL), [median (IQR)]	43 (35-54)	37 (31-46)	46 (37-58)	<.001
Platelet (10^3^/uL), [median (IQR)]	227.5 (183-283)	227 (183-292)	228 (183-280.5)	.443
Albumin (g/dL), [median (IQR)]	4.29 (3.99-4.53)	4.29 (4.03-4.53)	4.28 (3.99-4.53)	.995
Globulin (g/dL), [median(IQR)]	3.7 (3.5-4.0)	3.5 (3.4-3.8)	3.8 (3.5-4.2)	<.001
AST (U/L), [median (IQR)]	31 (23-44)	36 (25-56)	29 (22-40)	<.001
ALT (U/L), [median (IQR)]	29 (19-46)	38 (24-64.75)	25 (18-40)	<.001
ALP (U/L), [median (IQR)]	69 (57-85)	71 (59-85)	69 (56-85)	.111
GGT (U/L), [median (IQR)]	61 (49-75)	61 (49-69.75)	61 (49-75)	.833
Severe COVID-19 infection, n (%)	339 (34%)	118 (39.9%)	221 (31.6%)	.012

AST, aspartate aminotransferase; BMI, body mass index; COVID-19, coronavirus disease 2019; DBP, diastolic blood pressure; GGT, gamma-glutamyl transferase; HDL, high-density lipoprotein; HT, hypertension; DM, diabetes mellitus; IQR, interquartile range; MAFLD, metabolic dysfunction-associated fatty liver disease; SBP, systolic blood pressure.

**Table 2.** Association Between Risk Factors and Severe COVID-19 Infection, Using Binary Logistic Regression

Risk Factor	Severe COVID-19 Infection			
	Univariate		Multivariate	
	OR (95% CI)	*P*	Adjusted OR (95% CI)	*P*
MAFLD	**1.437 (1.084-1.905)**	**.012**	**1.912 (1.363-2.684)**	**<.001**
Age	**1.063 (1.053-1.073)**	**<.001**	**1.053 (1.042-1.064)**	**<.001**
Sex	0.556 (0.427-0.725)	<.001	0.395 (0.282-0.554)	<.001
BMI	1.026 (1.005-1.048)	.018	–	–
Alcohol	0.488 (0.346-0.686)	<.001	0.876 (0.547-1.404)	.583
Smoking	0.501 (0.322-0.778)	.002	0.484 (0.2690-0.872)	.016
DM	3.877 (2.762-5.411)	<.001	–	–
HT	2.701 (2.040-3.575)	<.001	–	–
Platelet	0.997 (0.995-0.998)	<.001	0.999 (0.997-1.001)	.250
Cholesterol	1.004 (1.001-1.007)	.008	–	–
TG	1.002 (1.000-1.004)	.038	–	–
LDL	1.004 (1.001-1.007)	.007	–	–
HDL	0.993 (0.984-1.002)	.114	–	–
Albumin	0.159 (0.113-0.222)	<.001	0.246 (0.171-0.355)	<.001
Globulin	1.028 (0.742-1.426)	.867	–	–
AST	1.020 (1.014-1.025)	<.001	–	–
ALT	1.008 (1.004-1.012)	<.001	–	–
ALP	1.003 (0.999-1.008)	.138	–	–
GGT	1.000 (0.999-1.001)	.658	–	–

Correlation risk factor: BMI, obesity, DM, HT, SBP, DBP, chol., TG, LDL, HDL, globulin, AST, ALT.

ALP, alkaline phosphatase; ALT, alanine transminase; AST, aspartate transminase; BMI, body mass index; COVID-19, coronavirus disease 2019; DM, diabetes mellitus; DBP, diastolic blood pressure; GGT, gamma-glutamyl transferase; HDL, high-density lipoprotein, HT, hypertension; LDL, Lipoprotein; MAFLD, metabolic dysfunction-associated fatty liver disease; OR, odds ratio; SBP, systolic blood pressure; TG, triglyceride.

**Table 3.** Association Between Risk Factors and Severe to Critical Illness of COVID-19 Infection in MAFLD Patient, Using Binary Logistic Regression

Risk Factor	Severe to Critical Severity of COVID-19 Infection							
	Univariate		Multivariate (NFS)		Multivariate (FIB-4)		Multivariate (FIB-8)	
	OR (95% CI)	*P*	Adjusted OR (95% CI)	*P*	Adjusted OR (95% CI)	*P*	Adjusted OR (95% CI)	*P*
Age	1.056 (1.039-1.074)	<.001	1.035 (1.010-1.060)	.005	1.037 (1.013-1.062)	.002	1.028 (1.003-1.052)	.023
Sex	0.762 (0.478-1.216)	.255	–	–	–	–	–	–
BMI	0.959 (0.923-0.996)	.031	0.973 (0.924-1.024)	.297	0.986 (0.941-1.033)	.555	0.968 (0.921-1.017)	.202
DM	1.866 (1.091-3.192)	.023	0.882 (0.436-1.786)	.728	0.982 (0.504-1.911)	.957	0.775 (0.376-1.595)	.448
HT	2.421 (1.478-3.967)	<.001	0.949 (0.490-1.839)	.877	0.954 (0.494-1.844)	.889	0.946 (0.493-1.815)	.867
Platelet	0.995 (0.992-0.998)	.002	0.997 (0.993-1.002)	.241	0.996 (0.992-1.000)	.065	0.998 (0.994-1.001)	.193
Cholesterol	1.000 (0.995-1.004)	.88	–	–	–	–	–	–
TG	0.999 (0.996-1.002)	.408	–	–	–	–	–	–
LDL	1.001 (0.996-1.006)	.663						
HDL	0.992 (0.975-1.009)	.357						
Albumin	0.167 (0.089-0.314)	<.001	0.249 (0.123-0.505)	<.001	0.216 (0.109-0.426)	<.001	0.252 (0.127-0.498)	<.001
Globulin	2.959 (1.382-6.333)	.005	2.862 (1.188-6.894)	.019	3.056 (1.260-7.411)	.013	2.798 (1.126-6.951)	.027
AST	1.005 (0.998-1.011)	.154						
ALT	1.002 (0.999-1.004)	.142						
ALP	0.999 (0.993-1.006)	.870						
NFS score	1.595 (1.361-1.868)	<.001						
NFS low	Ref		Ref					
NFS intermediate	4.047 (2.372-6.904)	<.001	1.924 (0.814-4.544)	.136				
NFS high	7.313 (3.205-16.687)	<.001	1.672 (0.402-6.962)	.480				
FIB-4 score	1.913 (1.511-2.424)	<.001						
FIB-4 low	Ref				Ref			
FIB-4 intermediate	3.066 (1.766-5.344)	<.001			1.665 (0.813-3.412)	.163		
FIB-4 high	5.378 (2.711-10.611)	<.001			1.187 (0.411-3.427)	0.751		
FIB-8 score	1.679 (1.388-2.032)	<.001						
FIB-8 low	**Ref**						**Ref**	
FIB-8 intermediate	**5.375 (1.976-14.621)**	**<.001**					**3.553 (1.143-11.043)**	**.028**
FIB-8 high	**15.115 (5.669-40.302)**	**<.001**					**5.458 (1.481-20.110)**	**.011**

BMI, body mass index; COVID-19, coronavirus disease 2019; DM, diabetes mellitus; MAFLD, metabolic dysfunction-associated fatty liver disease; NFS, nonalcoholic fatty liver disease fibrosis score; OR, odds ratio.

**Supplementary Table 1.** Correlation Between Each Pair of Risk Factors for Severe SARS-CoV-2 Illness in Hospitalized Patients

		Spearman’s rho	*P*
MAFLD	Age	-0.042	.183
	Sex	-0.035	.269
	BMI	.402	<.001
	Alcohol	-0.012	.699
	Smoking	.033	.296
	Diabetes	0.115	<.001
	Hypertension	0.046	.146
	Platelet	0.024	.443
	Cholesterol	0.156	<.001
	Triglyceride	0.168	<.001
	LDL	0.230	<.001
	HDL	-0.281	<.001
	Albumin	<0.001	.995
	ALT	0.238	<.001
Age	Sex	-0.003	.918
	BMI	0.005	.881
	Alcohol	-0.267	<.001
	Smoking	-0.134	<.001
	DM	0.332	<.001
	HT	0.499	<.001
	Platelet	-0.250	<.001
	Cholesterol	0.043	.191
	Triglyceride	0.003	.916
	LDL	0.037	.262
	HDL	0.013	.685
	Albumin	-0.389	<.001
	ALT	0.048	.129
Sex	BMI	-0.055	.085
	Alcohol	-0.199	<.001
	Smoking	-0.264	<.001
	DM	-0.033	.292
	HT	-0.011	.734
	Platelet	0.216	<.001
	Cholesterol	-0.097	.003
	Triglyceride	-0.138	<.001
	LDL	-0.167	<.001
	HDL	0.283	<.001
	Albumin	-0.073	.022
	ALT	-0.317	<.001
BMI	Alcohol	-0.115	<.001
	Smoking	0.026	.419
	Diabetes	0.143	<.001
	Hypertension	0.161	<.001
	Platelet	0.054	.088
	Cholesterol	0.235	<.001
	Triglyceride	0.275	<.001
	LDL	0.315	<.001
	HDL	-0.366	<.001
	Albumin	-0.060	.057
	ALT	0.322	<.001
Alcohol	Smoking	0.453	<.001
	Diabetes	-0.109	<.001
	Hypertension	-0.146	<.001
	Platelet	-0.067	.034
	Cholesterol	-0.010	.767
	Triglyceride	0.041	.213
	LDL	-0.017	.602
	HDL	0.006	.845
	Albumin	0.130	<.001
	ALT	0.025	.423
Smoking	Diabetes	-0.043	.180
	Hypertension	-0.072	.024
	Platelet	.001	.969
	Cholesterol	.029	.373
	Triglyceride	0.068	.037
	LDL	0.035	.281
	HDL	-0.065	.046
	Albumin	0.072	.023
	ALT	0.038	.229
Diabetes	Hypertension	0.370	<.001
	Platelet	-0.118	<.001
	Cholesterol	0.108	<.001
	Triglyceride	0.074	.024
	LDL	0.122	<.001
	HDL	-0.096	.003
	Albumin	-0.125	<.001
	ALT	0.086	<.001
Hypertension	Platelet	-0.115	<.001
	Cholesterol	0.086	.009
	Triglyceride	0.045	.167
	LDL	0.114	<.001
	HDL	-0.085	.010
	Albumin	-0.196	<.001
	ALT	0.089	.005
Platelet	Cholesterol	0.108	<.001
	Triglyceride	0.127	<.001
	LDL	0.068	.038
	HDL	0.031	.343
	Albumin	0.072	.022
	ALT	-0.038	.230
Cholesterol	Triglyceride	0.776	<.001
	LDL	0.909	<.001
	HDL	-0.369	<.001
	Albumin	-0.128	<.001
	ALT	0.252	<.001
Triglyceride	LDL	0.683	<.001
	HDL	-0.436	<.001
	Albumin	-0.136	<.001
	ALT	0.277	<.001
LDL	HDL	-0.650	<.001
	Albumin	-0.152	<.001
	ALT	0.299	<.001
HDL	Albumin	0.159	<.001
	ALT	-0.308	<.001
Albumin	ALT	-0.022	.492
